# Theranostic Nanomedicines for the Treatment of Cardiovascular and Related Diseases: Current Strategies and Future Perspectives

**DOI:** 10.3390/ph15040441

**Published:** 2022-04-01

**Authors:** Natasha Manners, Vishnu Priya, Abhishesh Kumar Mehata, Manoj Rawat, Syam Mohan, Hafiz A. Makeen, Mohammed Albratty, Ali Albarrati, Abdulkarim M. Meraya, Madaswamy S. Muthu

**Affiliations:** 1 Department of Pharmaceutical Engineering and Technology, Indian Institute of Technology (BHU), Varanasi 221005, India; natashamanners.phe19@itbhu.ac.in (N.M.); vishnupriya.rs.phe18@itbhu.ac.in (V.P.); ahisheshkm.phe15@itbhu.ac.in (A.K.M.); 2 Novartis Healthcare Private Limited, Hyderabad 500078, India; bitsmanoj@gmail.com; 3 Substance Abuse and Toxicology Research Center, Jazan University, Jazan 45142, Saudi Arabia; syammohanm@yahoo.com; 4 School of Health Sciences, University of Petroleum and Energy Studies, Dehradun 248007, India; 5 Pharmacy Practice Research Unit, Clinical Pharmacy Department, College of Pharmacy, Jazan University, Jazan 45142, Saudi Arabia; hafiz@jazanu.edu.sa (H.A.M.); ameraya@jazanu.edu.sa (A.M.M.); 6 Department of Pharmaceutical Chemistry, College of Pharmacy, Jazan University, Jazan 45142, Saudi Arabia; malbratty@jazanu.edu.sa; 7 Rehabilitation Health Sciences, College of Applied Medical Sciences, King Saud University, Riyadh 11451, Saudi Arabia; albarrati@ksu.edu.sa

**Keywords:** atherosclerosis, cardiovascular disease, personalized therapy, pharmaceutical nanomedicine, targeted delivery

## Abstract

Cardiovascular and related diseases (CVRDs) are among the most prevalent chronic diseases in the 21st century, with a high mortality rate. This review summarizes the various nanomedicines for diagnostic and therapeutic applications in CVRDs, including nanomedicine for angina pectoris, myocarditis, myocardial infarction, pericardial disorder, thrombosis, atherosclerosis, hyperlipidemia, hypertension, pulmonary arterial hypertension and stroke. Theranostic nanomedicines can prolong systemic circulation, escape from the host defense system, and deliver theranostic agents to the targeted site for imaging and therapy at a cellular and molecular level. Presently, discrete non-invasive and non-surgical theranostic methodologies are such an advancement modality capable of targeted diagnosis and therapy and have better efficacy with fewer side effects than conventional medicine. Additionally, we have presented the recent updates on nanomedicine in clinical trials, targeted nanomedicine and its translational challenges for CVRDs. Theranostic nanomedicine acts as a bridge towards CVRDs amelioration and its management.

## 1. Introduction

Cardiovascular is aword combining “cardio” implying the heart and “vascular” means blood vessels. CVRDs are chronic disorders of the heart and the vascular systems such as arteries, veins, and blood capillaries. CVRDs include both cardiovascular diseases and cardiovascular related diseases such as angina pectoris, myocarditis, myocardial infarction, pericardial disorder, thrombosis, atherosclerosis, hyperlipidemia, hypertension, pulmonary arterial hypertension, stroke, etc. [1]. Altogether, CVRDs are the leading causes of mortality and morbidity in developed countries, owing to their sedentary lifestyles [2]. Furthermore, obesity and diabetes, and related disorders, are linked with CVRDs, hence becoming a comorbid relation. Furthermore, a complication such as coronary artery obstruction is among the number one causes of death in adults that lead to thrombus plagues in the intima of the arterial wall, mainly in the carotid artery [3]. Cardiovascular diseases are prevalent in high-income countries such as the United States of America, but they have also become global issue. In 2016, a report demonstrated that 62.5 and 12.7 million people died due to cardiovascular diseases in India and America, respectively. Out of these deaths, most causes for deaths were observed as ischemic heart disease and stroke, and likely more will be in the future if serious actions are not taken [4].

The latest milestone in the treatment of CVRDs is the surgical implantation of coronary stents, which will be helpful in atherosclerosis and other coronary artery diseases. However, other medical managements of CVRDs rely on conventional medications such as beta-blockers, diuretics, hypolipidemic drugs, etc. The conventional medicines for the management of CVRDs are non-targeted drug delivery systems that can produce some potential side effects and toxicities. Developing targeted nanomedicine for CVRDs can minimize or eliminate these associated problems. Additionally, integrating targeted nanomedicine with diagnostic agents can detect and deliver the drug to the targeted site and provide a better understanding of the disease and its treatment processes [5]. For example, macrophages play a key role in the development and progression of atherosclerosis. Hence, designing targeted theranostic nanomedicine for imaging and targeted therapy of macrophage-associated pathological processes in the CVRDs [6]. Surgical implants suffer from critical problems such as infection at the implantation site, blood clots, and damage to blood vessels, whereas beta-blockers slow down the heartbeat and reduce the force of contraction, leading to the heart’s adaptation to a slower speed if taken regularly. Suddenly stopping medication of the beta-blocker increases the risk of a heart attack. Diuretics have the inherent disadvantages of triggering body ionic imbalances, such as lower sodium and potassium levels. Therefore, newer, safer, and more effective treatment modalities need to be developed to diagnose and treat CVRDs. The emerging theranostic nanomedicine modalities recently developed for diagnosing and treating CVRDs include nanoparticles, liposomes, metallic nanovesicles, etc. [7]. Therapeutic agents in theranostic nanomedicine include, synthetic or semisynthetic drugs, peptides, protein and genomic materials [8]. In contrast, commonly used diagnostic agents in theranostic nanomedicine include fluorescent dye [9] or quantum dots (optical imaging) [10], superparamagnetic iron oxide nanoparticles (magnetic resonance imaging) [11], radioactive nucleoid (nuclear imaging) [12] and heavy elements such as iodine [13] for computed tomography. Quantum dots possess numerous advantages over organic dye that include higher signal intensity, brightness, and photostability. In recent decades, nanomedicines have taken the world by storm for their application in the diagnosis and treatment of cancer. Researchers have recently emphasized theranostic aspects of nanomedicines on other illnesses and diseases such as CVRDs [14].

In addition, targeted nanomedicines are gaining momentum in the medical field because of their advancement in specificity-based therapy through targeting cells and cellular components. Targeted approaches have created advanced nanomedicine platforms for drug targeting and disease diagnosis at a nanoscale level [15]. Conventional treatments have lower efficacy, elevated systemic side effects, and a shorter drug half-life. For instance, an endogenous substance called urokinase acts as a thrombolytic agent. It is often used in various CVRDs, such as strokes, adjunctive therapy after angioplasty of an occluded artery, pulmonary embolism, etc. However, it has a major drawback of having a short half-life, which requires continuous administration to achieve a proper therapeutic window. Furthermore, it has been shown to cause excessive bleeding elsewhere in the body because it prevents platelets from aggregating properly to form blood clots under normal conditions. The use of urokinase encapsulated in a nanoformulation during targeted delivery was shown to result in an increase in half-life associated with a decrease in systemic toxicity, according to the findings [16]. Theranostic nanomedicine has demonstrated its potential role in diagnosing and treating the disease in preclinical studies. However, new advancement in drug delivery and diagnosis have been developed but still remains in their infancy stage for clinical application. Nanoparticulate and biological interaction are the key challenges associated with the clinical translation of theranostic nanomedicine. When a nanoparticle interacts with biomaterials, its possible toxicity or incompatibility might cause problems such as immunoreaction, inflammation, and other disorders. The harmful effects of nanomedicines are highly reliant on numerous aspects such as size, zeta-potential, and solubility. Hence, preclinical studies in numerous animal models will be helpful for addressing this issue. Additionally, academic research laboratories develop theranostic nanomedicine on a modest scale, focusing on emerging scientific and technological advancements. They are frequently aware of the technical obstacles that the industry faces while commercializing techniques. Increased coordination between labs and pharmaceutical industries is required to narrow this gap [17].

This review summarizes the use of different theranostic nanomedicines for diagnostic and therapeutic purposes in CVRDs. The past and ongoing clinical trials, including emerging techniques such as artificial intelligence, gene therapy, tissue engineering, etc., that have been utilized as a subsidiary tools, were also discussed. Additionally, the review provides a glimpse of challenges associated with the theranostic nanomedicine therapy of CVRDs.

## 2. Diagnostic Application of Targeted Nanomedicine in CVRDs

Early detection of any disease is the key to any treatment, as the higher progression of conditions can be fatal and life-threatening. Hence, with advancements in the medical field, nano-scaled contrasting agents have been employed in the early diagnosis of CVRDs. Contrasting agents such as quantum dots have mainly been used to capture fluorescence tomography images. Additionally, diagnostic agents such as 18F-crosslinked iron oxide (18F CLIO) are used for radioactive scans such as PET, SPECT, etc. In a few diagnoses, gadolinium chelated with diethylenetriamine pentetic acid (Gd-DTPA), iron oxide nanoparticles in magnetic resonance imaging (MRI) [18], gold or iodine-based nanoparticles in computed tomography (CT) imaging [19], gold nanoshells in optical coherent tomography, and colloidal nanobeacons in photoacoustic tomography have been reported [20]. There are also other multimodal techniques have been used, such as copper-CLIO in MRI, positron emission tomography (PET), and near-infrared fluorescence (NIRF) [7,21].

### 2.1. Quantum Dot-Based Imaging of CVRDs

In one study, a very stable CT-targeted contrasting agent was developed with lisinopril as the targeting ligand, and was further incorporated into citrate-coated gold nanoparticles. The results showed an image of the heart and lung regions indicating the targeting of an angiotensin-converting enzyme (ACE), which was overexpressed in cardiac and pulmonary fibrosis. It was reported that the developed nanoprobes would be very useful tools for monitoring cardiovascular pathophysiology using CT imaging. Furthermore, in vivo studies in mice depicted that lisinopril-thioctic acid conjugated nanoparticles are accumulated specifically in the lungs and heart due to higher density ACE receptors after intravenous administration and produced stable X-ray CT images of the lungs and heart [22]

### 2.2. Magnetic Nanoparticles-Based Imaging of CVRDs

The polymeric nanoparticles that carry contrasting agents are safer and better alternatives for producing high-resolution images than free contrasting agents. The developed targeted nanocolloids have demonstrated promising in vitro MRI contrast agents due to their higher relaxivity and detection sensitivity than non-targeted nanocolloids [23]. Additionally, with advanced technologies, medical devices are programmed for the rapid diagnosis of CVRDs. Bio-nanochips, made up of nanonets and quantum dots, produce results in minutes [24]. As diagnosis is emphasized in early biomarkers detection, it is necessary to identify biomarkers involved in CVRDs. These biomarkers include troponins, creatine k muscle/brain, B-type natriuretic peptide, myeloperoxidase, apolipoproteins, myoglobin, low-density, and high-density lipoprotein [25]. Furthermore, biomarkers were targeted by paramagnetic/fluorescent micellar nanoparticles in the ischemic or reperfused mouse model. Micellar nanoparticles were found to diagnose the onset of acute myocardial infarction by detecting the overexpressed extracellular matrix metalloproteinase inducer [26].

Currently, with the help of molecular imaging and MRI at the nanoscale, a non-invasive technique of diagnosis has been developed. This MRI allows better photon penetration in a specific nanometer range and the scattering of beams of light can be diminished to show better clarity with heightened sensitivity at detection [27]. Utilizing this technology, imaging of cardiomyocyte apoptosis incorporating magneto-optical nanoparticles can produce high-resolution in vivo images [28]. In a similar study, the diagnosis of cardiomyocyte apoptosis was made with annexin-labeled nanoparticles and it was observed that a low level of apoptosis in the myocardium indicated a healthy heart and the elevated apoptotic levels of cardiomyocytes revealed the heart abnormality. In conclusion, this study suggested developing a nanoplatform for cardiac diagnosis and developing novel anti-apoptotic heart failure therapies [29,30].

In atherosclerosis, various molecular imagining techniques have been reported, such as intravital microscopy, nanoparticle-enhanced molecular MRI, multicolor spectral CT, and fluorescence imaging; further quantification of blood vessel wall inflammation has also been imaged by PET. Many of these imaging techniques mentioned above are currently undergoing clinical trials [31]. Other cellular targets used as biomarkers in atherosclerosis include macrophages, cell adhesion molecules (CAMs), lipids such as low-density lipoprotein (LDL), high-density lipoprotein (HDL) and fibrin. Furthermore, increases in angiogenesis have been observed at the initial stages of atherosclerotic plaque formation, which serves as a good target for diagnosis. In one study, angiogenesis was evaluated by targeting a molecule called αvβ3 integrin, and it was visualized using an MRI scan [32]. In some studies, imaging techniques of thrombosis have been accomplished using atomic force microscopy through sizes of the thrombotic clots. This tool offers better resolution and clarity at a lesser range of 1 nm. In addition to this, it enabled the analysis of platelet activities and their points of activation in conjunction with the analysis of proteins involved in thrombosis [33]. In the early 2000s, the diagnosis of a thrombotic clot in vivo was also made by an MRI scan in canine models. It was fabricated with anti-fibrin monoclonal antibodies in conjunction with lipid-encapsulated perfluorocarbon nanoparticles comprising gadolinium-chelate as a targeting agent [34]. In another study, ultra-small superparamagnetic iron oxide nanoparticles in conjunction with fucoidan were reported to diagnose arterial thrombi by MRI visualization. Surface plasmon resonance imaging demonstrated that targeted nanoparticles binds to immobilized P-selectin in vitro. All intraluminal hypo-signals detected by MRI after injection targeted the nanoparticle, whereas none could be identified with non-targeted nanoparticles [35].

### 2.3. Radio-Imaging of CVRDs

Similarly, ligand-targeted 19F perfluorocarbon nanoparticles have been used to visualize and examine unstable in vivo atherosclerotic lesions. In this study, the nanoformulation includes an exquisite contrast agent for the paramagnetic property. The formulation consisted mainly of lecithin encapsulated liquid perfluorocarbon with a massive amount of chelated gadolinium, which was located within the bounding lipid. The targeting property of formulated particles with distinct monoclonal antibodies showed nanoparticles localization for the MRI signal amplification in imaging areas with minor or no opposing blood pool signal [36].

## 3. Therapeutic and Theranostic Applications of Targeted Nanomedicine in CVRDs

Nanocarriers are loaded with therapeutic agents which are directly transported to the target site, either by passive or active targeting. For passive targeting, the nanomedicine reaches the target site employing highly perfusable cells. In active targeting, the nanomedicine is conjugated with site-specific molecules or cell-specific targeting ligands [15,37]. A study was carried out with liposome-encapsulated amiodarone showing better therapeutic efficacy with lesser side effects in hypotension. This is an excellent example of how nanoformulation of cardiac drugs can aid in better therapy of CVRDs [38]. Moreover, liposomal vesicles containing surface charge due to their compositions can also be utilized for molecular targets. For example, cationic lipid such as dioleoylphosphatidylethanolamine (DOPE) were used to prepare liposomes that carried a positive charge and showed a greater affinity towards negatively charged targets such as nucleic acids, tumor cells, intestinal mucosa, etc. [39]. Different targeting approaches for nanocarrier-based therapy for cardiovascular and cardiovascular related diseases are presented in Table 1 and Table 2.

### 3.1. Nanomedicine as a Drug Delivery System for Cardiovascular Diseases

#### 3.1.1. Nanomedicines for Angina Pectoris

Angina pectoris is a health condition occurring when an insufficient supply of oxygenated blood reaches the myocardial tissues. A potassium channel opening drug-like nicorandil is given in the case of angina pectoris. Polymeric nanofibers containing nicorandil were developed using the electrospinning method that utilizes Vitamin B_12_ and a mixture of hyaluronic acid and polyvinyl alcohol as polymeric ingredients, which counter the adverse consequences associated with the oral administration of nicorandil. The pharmacokinetics study suggested that polymeric nanofibers have a longer plasma half-life of 3.7 h than the marketed preparation of nicorandil with a 0.99 h plasma half-life. This may be due to the greater therapeutic absorption of developed nanofibers and their sustained release characteristics. After sublingual delivery of nicorandil, the histopathology examination found no signs of mucosal ulcers [70]. This condition can occur concurrently with obstructive coronary artery disease or myocardial hypertrophy. Additionally, it was found that low oxygen levels can lead to ischemia of the heart [71].

When angina occurs, patients feel a sharp pain in their chest, shoulder, neck, arm, and back due to blocked or narrowed arteries that deprive the blood supply to their cardiac muscles [40]. There are two types of angina pectoris: the classic form of anginaand stable angina. The other is a variant form of angina or unstable angina or prinzmetal’s angina. In some cases, it occurs when the patient is engaging in physical activities resulting in increased cardiac workload, i.e., the classic form of angina. In the variant form of angina, attacks can occur even when the patient is at complete rest without physical activity [72]. Conventional treatments include the administration of low-cost nitrates such as nitroglycerin. However, it is short-lasting and undergoes the first-pass metabolism. Hence, longer-acting nitrates such as isosorbide dinitrate have been used in treatment [73]. It was found that drugs such as ivabradine in tablet form lowered the heart rate, but their pharmacokinetic property were poor. A study reported that ivabradine was loaded in polymeric nanoparticles and the oral bioavailability and the half-life of ivabradine were improved. It was reported that the first-pass metabolism of ivabradine was reduced by the enzyme cytochrome P450 3A4 (CYP3A4) [40].

In another study, verapamil was used with low bioavailability and a high first-pass effect. Verapamil is a calcium channel antagonist, and it is very efficacious in managing hypertension, angina pectoris, and other diseases. However, low bioavailability and low cellular uptake were reported as the drawbacks of this drug. In non-targeted management of angina pectoris, a study was reported where verapamil-dextran was incorporated in nanostructured lipid carriers. Furthermore, in vitro cellular uptake in Caco-2 cells was increased by ~2.47-fold compared to non-formulated free verapamil. The results showed an enhanced cellular uptake of verapamil [41]. Mostly anti-anginal drugs effectively control angina, but they have low bioavailability, e.g., nicorandil. These drugs are good vasodilators that act on potassium (K+) ATP channel opening. Researchers have incorporated nicorandil into various nanocarriers to overcome this drawback of low bioavailability. One such carrier was reported, i.e., a biocomposite polymeric nanofiber of nicorandil, which was delivered sublingually to reduce mucosal ulceration and successfully improvethe oral drug bioavailability [70].

Minimal advancement was observed for active targeting therapy in angina pectoris. A study was reported with cardiomyocyte-enabled targeting by radiofrequency ablation in rats. This study showed the targeting effect of amiodarone hydrochloride (ADHC) loaded liposomes with the help of radiofrequency ablation. They found that the ADHC concentration in the heart was 4.1-fold higher in the case of ADHC liposomes after 20 min post-injection [74]. Other potential targets reported for managing these cardiovascular diseases include carnitine-palmitoyltransferase-1 (CPT-1), carnitine-palmitoyltransferase-2 (CPT-2), carnitine-acylcarnitine translocase, gamma-butyrobetaine hydroxylase, etc. These inactivated targets were found to change the process of fatty acid oxidation into glucose oxidation, which modified the overall cardiac workloads [75].

#### 3.1.2. Nanomedicines for Myocarditis

Inflammation is an act of the body’s defense mechanism, arising due to various factors such as virulent or bacterial attack, physical agents, excess ROS generation, hypoxia, etc. The nanomedicines have an edge over inflammation therapy as their small size can be engulfed by inflammatory cells such as macrophages. It can either cause inhibition of the expression of inflammatory modulators or act as genosensors [55,76,77]. Additionally, various approaches to facilitate nanocarriers for myocarditis were attempted such as ligand decoration to mimic natural cell adhesion molecules (CAMs), anti-vascular cell adhesion molecule, lymphocyte function-associated antigen-1, anti-oxidized low-density lipoprotein receptor, etc. CAMs act as a biomarker of inflammation that is overexpressed and increased with immune cellular adhesion. Macrophages, as well as leukocytestarget inflamed endothelium irrespective of the injury. Hence, carriers that mimic biological molecules tend to trail after leukocyte tropism and precisely deliver cargo to the diseased area [77]. Other inflammatory targets are chemokine; these are minute chemotactic cytokines whose roles facilitate the recruitment of white blood cells to inflamed tissues. They are of various types, such as CCL2/CCR2 chemokine, which give rise to monocyte inflammatory recruitment in atherosclerosis and infarcted heart. Lipidic nanoparticles were reported with a small interfering RNA against C-C chemokine receptor type 2 (CCR2) that reduced inflammatory monocyte recruitment and overall inflammation in ischemia and atherosclerosis [78]. Another chemokine being targeted is interleukin 8 (CXCL8), over-expressed in inflamed conditions by the endothelial cells. Furthermore, iron metal-organic framework nanoparticles known as materials institute lavoisier-89 or nanoMIL-89 have shown an anti-inflammatory effect by reducing chemokine CXCL8 from human pulmonary artery endothelial cell lines. The microscopic cellular uptake study confirmed the internalization and packaging of nanoMIL-89 in endocytic vesicles and suggested their transfer to daughter cells during mitosis [42,78].

#### 3.1.3. Nanomedicines for Myocardial Infarction

Ischemia in myocardial cells happens when there is less oxygen supply in the blood from coronary blood flow, which leads to myocardial infarction. It is also known as a heart attack that leads to heart muscle injuries. When ischemia stops and blood oxygen level becomes normal, all abnormal behavior will drastically cease. However, if the oxygen level does not return to normal within an hour or so, it will lead to myocardial necrosis. If ample blood flow is returned within 15 min, myocardial infarction will cease to exist, but the myocardial cells will take time for normal function [42,79]. At myocardial infarction, inflammatory responses are one step behind, with releases of cytokines and leukocytes in the area. The higher production of tumor necrosis factor-alpha (TNF-α), interleukin-1 (IL-1), and interleukin-6 (IL-6) have been linked with myocyte death, myocardial injury and the healing processes, whereby blocking all these factors can lead to therapeutic action. Nanoparticles containing miR199a-3p were engulfed in membranes from engineered macrophages with TNF-αR, IL-1βR and IL-6R that contributed to cardiomyocyte cell proliferation. This engineered macrophage was administered intravenously in Balb/c mice that had been subjected to hypoxic conditions. The finding was a reduction in inflammatory cells in the infarcted region and increased cell proliferation abilities [43]. Another instance of targeting inflammatory monocytes/macrophages is using a PPARγ agonist-like pioglitazone in the myocardial injury site. It was encapsulated in bioabsorbable poly (lactic-co-glycolic acid) (PLGA) polymeric nanoparticles, which were taken up by monocytes and macrophages via phagocytosis. This dramatically enhanced the accumulation of PLGA nanoparticles in the ischemia-reperfusion myocardium through enhanced permeability of blood vessels in adult male C57BL/6J mice. Additionally, previous studies suggested that free pioglitazone has no efficacy when administered via a different route, such as the intraperitoneal route. Pioglitazone nanoparticles were found to suppress the production of Ly6C^high^ inflammatory monocyte and inflammatory gene expression in the myocardial ischemia-reperfusion hearts.

Additionally, it has been shown to decrease the mortality rate by 37% compared to the saline-treated group by 58% in a myocardial infarction survival study on mice [44]. Specific peptides have also been shown to inhibit the synthesis of reactive oxygen species (ROS), reduce the inflammatory response, and stimulate angiogenesis. Adenosine peptide was used as a prodrug along with synthesized atrial natriuretic peptide, which was loaded into lipid nanocarriers to manage myocardial infarction. Additionally, size and tissue distribution in ischemic myocardium were seen in Sprague Dawley rats after subsequent intravenous injections. They exhibited more promising results than their non-nanocarrier counterparts. It was found that half-life of the nanoencapsulated adenosine was 3.4 h longer than free adenosine [45]. Growth factors that stimulate angiogenesis were encapsulated in nanocarriers loaded hydrogel for sustained delivery. The developed ployglutamic acid complex VEGF nanomedicine has more than 99% encapsulation efficiency and sustained vascular endothelial growth factor (VEGF) release up to 35 days after incorporation into a biocompatible hydrogel. The nanoencapsulated VEGF was delivered to the targeted area in a constant way to achieve angiogenesis of ischemic myocardial cells [80].

Traditional medicines have also found their way into the nano-medicinal field in a new context, e.g., traditional Chinese medicines. Natural compounds used such as salvianolic acid B and ginsenoside Rg 1 in the accurate and well-combined ratio were proven to be working well with improving myocardium structure and ventricular function compared to their counterparts. These two compounds were incorporated into a lipid-polymer hybrid nanocarrier with an arginyl-glycyl-aspartic acid (RGD) peptide ligand as the targeting ligand for integrin αvβ3 of activated endothelial cells. Furthermore, in vivo infarct therapy study depicted that RGD functionalized nanoparticles reduced the infarct size by ~1.85-fold compared to the free drug [46]. Similarly, insertion peptide was demonstrated to be utilized as a targeting moiety in the region of low extracellular pH. An insertion peptide acted as an unstructured peptide in the peripheral lipid layer; however, it started insertion in a slightly acidic medium. In a targeting efficacy study, fluorescent dyes and fluorescently labeled liposomes coated with this pH-sensitive peptide were utilized as imaging agents. The low pH insertion peptide-coated liposomes were reported with many benefits, such as drug transport moiety to risky zone [81]. Nanosized particles undergo various risk of opsonization by macrophages and other scavenger cells. To overcome this natural phenomenon, the surface of the nanoparticles was attached with single or additional polyethylene glycol (PEG) chains covalently to a drug moiety by the process of PEGylation. Alternatively, the opsonization of nanosized radix ophiopogonin polysaccharide was seen as a natural fructan with a special anti-myocardial ischemic activity. In addition, it showed an affinity to accumulate in the heart of myocardial ischemia-induced rats owing to the enhanced permeability and retention (EPR) effect. The mono-PEGylation of this fructan was carried out and showed enhanced therapeutic effects such as increased vascular proliferation, better delivery of the drug to the nonperfused necrotic zone, and lower morbidity rate in rats [47]. Paul et al., recently developed a nano-complex formulation for myocardial therapy that had graphene oxide and vascular endothelial growth factor along with pro-angiogenic gene. It was observed that low modulus methacrylated gelatin (GelMA) could effectively transport a nanocomplex of polyethyleneimine (PEI) linked with graphene oxide (fGO). Furthermore, the VEGF pro-angiogenic gene was investigated for myocardial treatment using injectable hydrogels. In an in vivo study in the rat model a significant increment in the myocardial capillary density at the periinfarct injection site and reduction in the scar area were observed in the myocardial infarcted heart compared to the control group [82].

#### 3.1.4. Nanomedicines for Pericardial Disorder

A few pericardial disorders have been classified under conditions such as excessive proliferation and stent restenosis after open-heart surgery. They are caused by the insertion of a drug eluting stent (DES) and inflammation of the double-walled pericardium. The former disorder was studied and tackled using patches that elude rapamycin nanoparticles in a sustained manner. It was found that this pericardial patch reduced the thickening of endothelial cell proliferation after open-heart surgery and stent restenosis [83]. Similarly, after the cardiac operation, the usage of supramolecular polymeric hydrogel for post-surgical care of the pericardial covering was studied for effective wound healing [84].

#### 3.1.5. Anti-Thrombotic Nanomedicines

**Figure 1:**
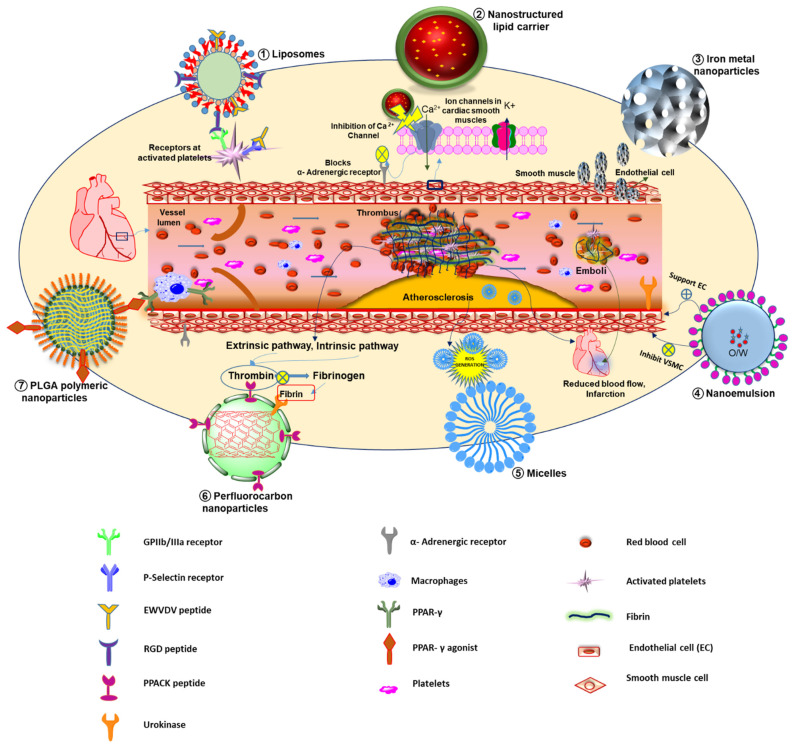
Schematic illustration of different principles involved in nanoparticles mediated therapy for CVRDs: (1) liposomal vesicle decorated with RGD and EWVDV peptide for targeting the GPIIb/IIIa and P-selectin receptors present on activated platelets for inhibiting thrombus formation, respectively, (2) verapamil encapsulated nanostructured lipid carrier shows higher cellular uptake and inhibit L-type calcium channel consequently antagonize α-adrenergic receptor, (3) iron-based porous metallic nanoparticles shows uptake by pulmonary arterial endothelial and smooth muscle cell, (4) 17-β estradiol and C6-ceramide loaded nanoemulsion inhibits VSMC proliferation, supports EC proliferation and cease restenosis, (5) andrographolide loaded self-assembled (PEG-PES) ROS responsive micelles render benefit of ROS generation associated with atherosclerosis therapy, (6) PPACK and urokinase decorated perfluorocarbon nanoparticle targets the coagulation cascade and inhibits thrombus formation, (7) PPAR-γ agonist containing PLGA nanoparticles that interacts with PPAR-γ receptors present on macrophages and provides cardio-protection in myocardial infarction.

**Figure 2:**
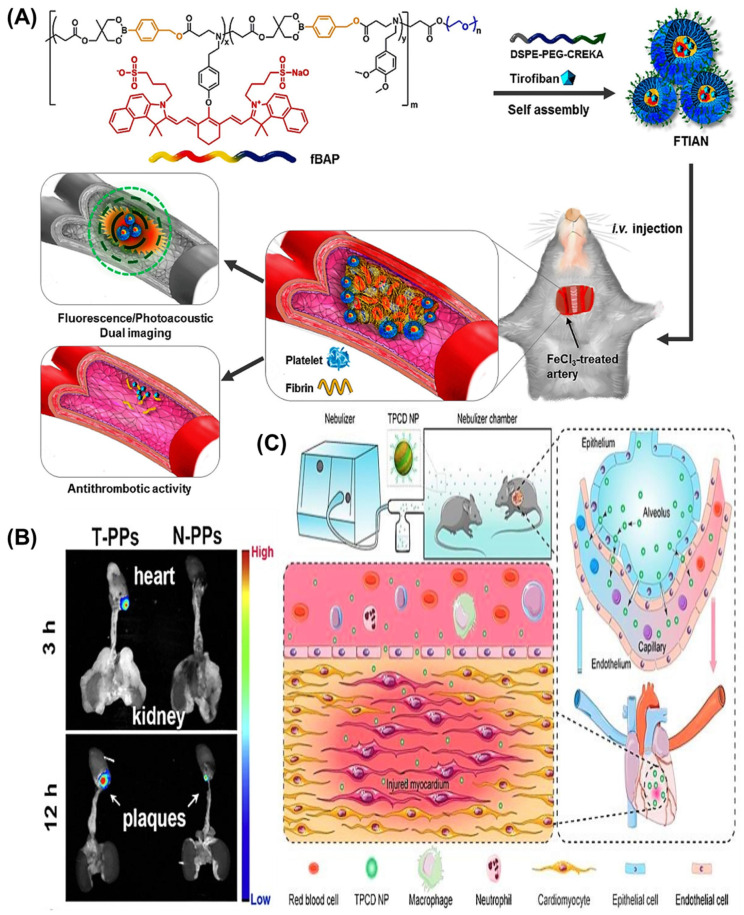
Theranostic nanomedicine for diagnosis and therapy of cardiovascular and related diseases. (**A**) A schematic representation of FTIAN as a fibrin targeted and H_2_O_2_ responsive nanoparticles as theranostic for thrombosed vessel. Reproduced with permission from ref [54], scheme 1 (ACS^®^ 2017). (**B**) Ex vivo fluorescence imaging of the aortas at different time points after mice received tail vein injections of AF750-SCR loaded nanomedicines. Reproduced with permission from ref [91] Figure 6. (ACS^®^, 2019). (**C**) Schematic illustration of in vivo targeting of the injured myocardium via inhalation of TPCD NPs. Reproduce with the permission from ref [48], Figure 1. (Ivyspring^®^ 2021). fBAP: Fluorescent dye-conjugated boronate antioxidant polymer; FTIAN: Fibrin targeted imaging and antithrombotic nanomedicine; CREKA: Pentapeptide Cys-Arg-Glu-Lys-Ala; FeCl_3_: Ferric chloride; DSPE: 1,2-distearoyl-sn-glycero-3-phosphoethanolamine; PEG: Poly ethylene glycol; NPPs: Non targeted polyplexes; TPPs: Targeted polyplexes; TPCD: A ROS scavenging material.

Thrombosis obstructs the flow of blood, leading to ischemia and myocardial infarction. It happens when an injury occurs at the atherosclerotic plaque, triggering platelet aggregation at the luminal site, resulting in rapid thrombus growth along with the development of a fibrin network that stabilizes platelet-rich thrombus [85,86]. Thrombus lysis can be induced by thrombolytic agents such as tissue-type plasminogen activator (tPA) that activate plasmin. Additionally, a serine protease could dissolve fibrin contained in clots [87]. An earlier study achieved accelerated thrombolysis in rabbits using streptokinase encapsulated in liposomes with water-soluble polymers. The occlusions were reduced with liposomal formulation than free streptokinase with multiple episodes of reocclusion and subsequent reperfusion. Compared to free streptokinase (74.9 ± 16.9 min.), drug encapsulated liposomes (19.3 ± 4.6 min.) and water-soluble polymers (7.3 ± 1.6 min.) have shown lesser reperfusion times [49]. The lipid bilayer vesicles are derived from a platelet bound with active integrin GPIIb-IIIa, P-selectin, GPIb-type receptors, thrombospondin, and C-X-C type chemokine and thrombin receptors. Specifically, these platelet-derived vesicles can be directed to the thrombus via binding to active platelet integrin GPIIb-IIIa and P-selectin. The chain reaction causes the vesicle to destabilize and relieves the thrombolytic payload that was activated by the clot-relevant enzyme, i.e., phospholipase-A2 [50]. The principles involved in nanoparticles mediated targeted therapy of CVRDs are presented in Figure 1. Targeted liposomal nanocarriers have been exploited with RGD peptide decoration that employs a higher affinity towards active platelet integrin GPIIb-IIIa receptors. RGD peptide ligand inactivates integrins GPIIb-IIIa, thus platelet adhesion and aggregation were prevented [88,89]. Conformation constraint of peptides such as cyclic RGD which was reported with liposomes that bonded to activated platelets considerably more than linear RGD-liposomes [90]. Semi-permeant perfluorocarbon core nanoparticles having D-phenylalanyl-L-prolyl-L-arginyl chloromethyl ketone as a targeting moiety have been reported to inactivate thrombin. The thrombin is a promoter of procoagulant factors synthesis, leading to endothelial disruption, coagulation, inflammation, and plaque expansion [51]. Another chitosan-coated magnetic theranostic nanoparticle encapsulating tissue plasminogen activator was developed by employing superparamagnetic properties. It ensures that the activity was altered externally by utilizing a magnetic field gradient. Furthermore, magnetization was discontinued once the magnetic field was deactivated. Overall this formulation has proven its efficacy as it requires only one-fifth of the regular dose of tPA to produce its thrombolytic activity [52]. In addition to this, a newer thrombus-targeted fibrinolytic agent was developed with theranostic and multifunctional properties. The study was based on crosslinked dextran-coated iron oxide (CLIO) nanoparticles with recombinant tissue plasminogen activator (r-tPA) conjunction that targets fibrin and activated factor XIII. It was reported to show theranostic capabilities and a high affinity towards clots; hence exhibiting its multipurpose tag [53]. To conserve the integrity of the blood vessels, generally, a thrombus (blood clot) forms in wounded blood vessels. It reduces the blood flow resulting in the death of cells fed by the arterial blood flow, which is a principal cause of various life-threatening cardiovascular disorders. Recently, Kang et al., developed fibrin-targeted theranostic nanoparticles that thrombosed blood vessels and simultaneously inhibit thrombus formation. The developed nanoformulation decreased the synthesis of tumor necrosis factor-alpha (TNF-α) and soluble CD40 ligand (sCD40L) in stimulated platelets. Additionally, it prevented the production of H_2_O_2_, suggesting its inherent antioxidant, anti-inflammatory, and antiplatelet action. They selectively attacked the obstructed thrombus in an animal model of ferric chloride (FeCl_3_)-induced carotid thrombosis and significantly increased the fluorescence/photoacoustic signal. The tirofiban-loaded nanoparticles were used at a concentration of 80 µg/kg in mice. They showed 10 and 7 folds higher bleeding time and blood loss, respectively which confirmed the significant suppression in thrombus development (Figure 2A) [54]. Research trends and available preclinical and clinical data suggested that theranostic nanomedicines are promising candidates for imaging and treatment of cardiovascular diseases.

### 3.2. Nanomedicine Approach in Cardiovascular-Related Diseases

#### 3.2.1. Anti-Atherosclerotic Nanomedicines 

**Figure 3:**
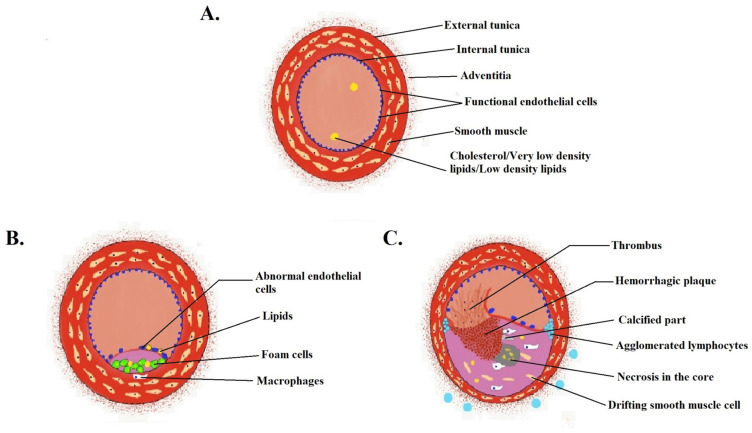
Pathophysiology of atherosclerosis. (**A**) The normal arteriole wall can be seen with fewer lipids in circulation. (**B**) Accumulation of lipids, foam cells, and macrophages leads to atherosclerotic plaque formation. (**C**) The latter stage of atherosclerosis leads to thrombus formation and necrosis in the plaque core which leads to different complications such as thrombosis and stroke.

Atherosclerosis is a condition that arises because of the excess accumulation of low-density lipids in the intima of arteries, forming hardened plaques. The main trigger of this chronic condition is an excess concentration of cholesterol (CH) in the blood that results in an alteration of arterial endothelial permeability that leads to lipids migration from the blood to the arterial wall [92]. The pathophysiology of atherosclerosis and thrombosis is depicted in Figure 3. Studies have revealed that inflammation and oxidative stress play a vital part in the aggravation of atherosclerosis. A conventional, poorly soluble anti-inflammatory agent, i.e., andrographolide, which inhibits the nuclear factor (NF)-κB signaling pathway, was loaded in PEG-poly(propylene sulphide) micelle and delivered passively to the drug target. It was found that the amphiphilic nanomicelle drug delivery and its efficacy were increased [55]. In some studies, anti-inflammatory classes of drugs such as steroidal drugs were used locally to treat atherosclerotic lesions. For example, glucocorticoids such as prednisolone may cause adverse effects such as dyslipidemia, glucose intolerance and hypertension if used systemically. It was minimized with the usage of nanoencapsulated prednisolone for targeted delivery at the atherosclerotic lesion. A similar study was carried out by another group of scientists where prednisolone phosphate was encapsulated in long circulating liposomes and showed the anti-inflammatory property of prednisolone but gave an insight into atherosclerotic imaging and diagnosis. The developed prednisolone liposomes have improved pharmacokinetics compared to the free drug in human control clinal trial [93]. In another report, prednisolone phosphate-loaded long-circulating liposomes were developed and were shown to increase the therapeutic outcome within two days of treatment that lasted for a week. Usually, anti-inflammatory agents are given weeks or months in advance to patients to reduce inflammation. Here, they developed a formulation that quickly reduced atherosclerotic inflammation [56].

Anti-inflammatory agent Interleukin-10 (IL10) is used in the treatment of clinical diseases such as rheumatoid arthritis, inflammatory bowel disease, psoriasis, chronic hepatitis C and atherosclerosis. However, direct administration of IL10 is not recommended for various reasons, due to severe side effects, such as a reduction in platelet count, transient neutrophilia, monocytosis, and lymphocytopenia and poor in vivo stability (shorter half-life and rapid clearance). Therefore, a study was conducted for targeted delivery in which IL-10 was encapsulated in chitosan nanoparticles, and cyclic arginine-glycine-aspartic acid (cRGD) peptide was used as a targeting ligand. The result showed an increase in the half-life of IL-10 with better serum concentration and a slower clearance rate than free IL-10. Furthermore, the result showed reactive oxygen species (ROS) such as superoxide anion (O2•−) to form peroxynitrite (ONOO-) was up-regulated at the atherosclerotic plaque site, which acted as a significant marker for macrophages to infiltrate for atherogenesis [57]. In another study, anti-inflammatory IL-10 was targeted at atherosclerosis plaque using liposomes in conjugation with cRGD. The results showed a lowering of ROS level and the inflammation factor, i.e., TNF-α in RAW 264.7 cells [94].

In addition to the inflammation process, elevated angiogenesis is a typical process in atherosclerosis. The angiogenic expansion increases plaque size, bleeding and lesion instability. An antiangiogenic agent such as fumagillin was combined with αvβ3 integrin-targeted paramagnetic nanoparticles to quantify and inhibit angiogenesis in atherosclerotic plaque in hyperlipidemic rabbits [58]. Moreover, genetic therapies have been developed using nanomedicine loaded with coding or non-coding genetic materials such as DNA or RNA for targeted delivery. In atherosclerosis, genetic materials such as microRNAs (miR-33, miR-21, miR-143, miR-145) were used to target myocardial smooth muscles, which reduced the production and accumulation of low-density lipids [95]. In another study, two diverse peptide sequences of miRNA inhibitors were incorporated into polyelectrolyte complex micelles. The first peptide, arginine-glutamic acid-lysine-alanine (REKA) was loaded into a micellar system and targeted atherosclerotic lesions in a mouse model. In contrast, the second peptide, valine-histidine-proline-lysine-glutamine-histidine-arginine (VHPKQHR), was recognized through phage display and targeted vascular endothelial cells via the VCAM-1 [96].

Furthermore, Mlinar et al., reported a VCAM-1 targeted delivery using self-assembled peptide amphiphilic micelles with a targeting peptide N-terminal cysteine (CVHPKQHR), and utilized cyanine 7 near-infrared dye as the labeling agent at near-infrared spectrum. It was observed that targeted micelles have higher binding and accumulation efficiency to murine aortic endothelial cells compared to non-targeted micelles. It showed profound theranostic activity at various stages of atherosclerotic plaque development [97].

Recently, 3-D nanoparticles are gaining momentum as nanocarriers available for medical imaging, ocular fluid replacement, blood replacement and liquid breathing, including the potential for targeted delivery, e.g., perfluorocarbon-based carriers containing perfluoro-chemicals in the core of the lipid encapsulated emulsion. This has been extensively studied to diagnose ruptured plaque and treated with drugs followed by angioplasty [98,99]. Atherogenesis is the leading cause of cardiovascular-related disorders that endanger global health due to structural changes and malfunctions of the endothelium. Regrettably, a practical approach for treating atherosclerosis is still distant from meeting clinical needs. The significant aspects that facilitate the formation of atherosclerosis are dyslipidemia and chronic inflammatory processes. The amplification of the pro-atherosclerotic component’s monocyte chemotactic protein 1 (MCP-1) and interleukin-6 (IL-6) was observed in plaques. Recently, Wu et al., designed a CD36 antibody-modified small interfering RNA (siRNA) nanoformulation based on the mPEG-PAsp-(g-PEI) vector for atherosclerosis treatment. In vitro and in vivo experiments showed that the produced siRNA nanoformulation targeted macrophages, and decreased CD36 activity, prevented IL-6 and MCP-1 overexpression. Subsequently, developed nanoformulation lowered foam cell production and relieved atherosclerotic pathology. Ex vivo fluorescence imaging of the aortas at different time points after mice received tail vein injections of AF750-SCR loaded nanomedicines Figure 2B [91].

Macrophages have displayed a crucial role in the development and progression of atherosclerosis and their local inflammatory responses. As macrophages are key immune cells present in the plaques, their number increases during atherosclerosis progression. Researchers have shown an interest in targeting the macrophages to attenuate the atherosclerotic progression and stabilizing the existing plaques. Recently, Zang et al., reviewed various nanoparticles for the passive and active targeting of macrophages in atherosclerosis. Passive targeting was achieved by using nanocarriers such as PLGA, magnetic nanoparticles, redox-sensitive micelles, β-cyclodextrin, etc., whereas active targeting was achieved by functionalization of the targeting ligand to the nanoparticle surface that includes mannose, folate, cRGD, ferritin, macrophage targeting peptide, hyaluronan and platelet membrane, etc. Passive targeting has improved the stability, controlled the release and prolonged the action of loaded therapeutics. In contrast, targeting has eliminated the toxicity associated with pure drug and non-targeted nanomedicine due to the site-specific release of the loaded therapeutics [100]. Nahrendorf et al., designed a magnetofluorescent theranostic nanoparticle labeled with PET tracer ^64^Cu for the imaging of inflammatory atherosclerosis. It was observed that after 24 h of intravenous administration of the labeled nanoparticles in mice with a low level of apolipoprotein E depicted high plaque load in computed tomography imaging. Furthermore, PET-CT and MRI studies suggested that the developed nanoparticle was capable of directly detecting macrophages in the atherosclerotic plaques ^64^Cu-TNP-based PET-CT imaging of the atherosclerotic mice model [101]

**Figure 4:**
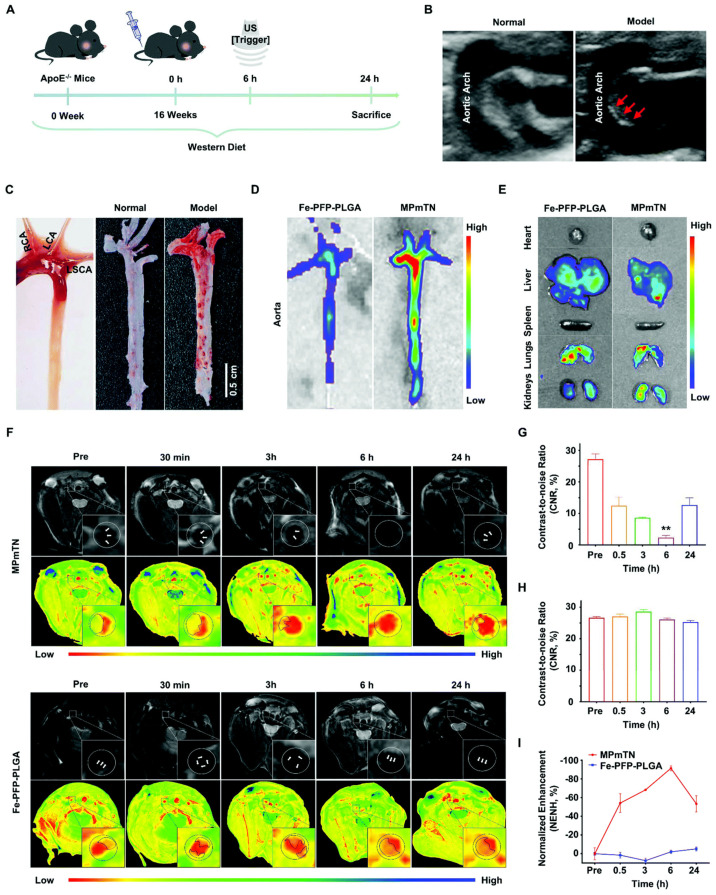
Illustrated in vivo targeting and imaging ability of Fe-PFP-PLGA nanoparticles and MPmTNs. In vivo imaging (**A**) time duration of the experiment, (**B**) aortic arch, (**C**,**D**) isolated arteries lumen, (**E**) isolated organs and (**F**) atherosclerotic plaque. (**G**,**H**) contrast to noise ratio and (**I**) % normalized enhancement of aortic plaques at different time point (** *p* < 0.01). Reproduced with permission from ref [59], Figure 6. (American Chemical Society^®^ 2021).

In a study, Gao et al., developed theranostic nanoparticles containing Fe_3_O_4_ and perfluoropentane with surface functionalization of plaque-targeted peptides (PP1) and cRGD for ultrasound/magnetic resonance imaging and ultrasound-assisted atherosclerosis treatment. The developed nanoparticles were efficiently able to target scavenger receptor-A (SR-A) and glycoprotein (GP) IIb/IIIa expressed on macrophages and activated platelet by binding through PP1 and cRGD, respectively. MPmTN and Fe-PFP-PLGA NPs represent targeted and nontargeted nanoparticles, respectively. Thus, the developed nanoparticle promotes macrophages apoptosis and the breakdown of the activated platelet; the same was confirmed in an in vivo study. Figure 4 illustrates in vivo targeted imaging of the aortic arch (B), isolated arterial lumen (C and D) and atherosclerotic plaque (F) by using developed nanoparticles [59]. Hence, theranostic nanomedicines have depicted promising outcomes in preclinical studies for detecting and treating atherosclerosis.

#### 3.2.2. Anti-Hyperlipidemic Nanomedicines

Hyperlipidemia is a condition in which serum lipids rise to an abnormal level. Lipids such as fatty acids, CH, CH esters, phospholipids, and triglycerides pose a risk in CVDs. It is a well-known fact that hyperlipidemia is one of the significant causes of atherosclerosis and atherosclerotic plaque, arousing conditions such as stroke, myocardial infarction and peripheral vascular disease. The higher lipid concentration in the blood leads to the risk of contracting atherosclerosis [102]. The harmful nature of lipids depends on their fatty chain structure and size. For example, low-density lipoproteins (LDL), triglycerides (TG) and very-low-density lipoproteins (VLDL) have smaller chain structures, sizes and molecular weight. It has been found that an increased level of these low molecular weight lipoproteins causes hyperlipidemia [103]. One study observed that ingestion of chitosan nanoparticles could induce a hypolipidemic effect in rats due to the better cholesterol-binding capacity of chitosan nanoparticles compared to chitosan supplements [104]. The chitosan nanoparticles were loaded with hypolipidemic agents such as poorly soluble statins. The simvastatin loaded in chitosan nanoparticles produced a synergistic hypolipidemic effect due to solubility enhancement in acidic pH, increasing the drug’s absorption and bioavailability. In addition, chitosan nanoparticles provided a sustained release by binding to mucus with self-swelling and gel formation [60].

Moreover, HDL is a cardioprotective agent due to its ability to facilitate reverse cholesterol transport and hinder inflammation at the plaque site. Therefore, the overall reduction in atherosclerotic plaque and its vulnerability were observed [105]. Besides this, reconstituted HDL (rHDL) was synthesized to mimic the endogenous form of HDL. It was composed of phospholipids, apolipoproteins, triglycerides, free cholesterol and rHDL, which were fitted with targeting ligands to evade reuptake by the liver and facilitated by scavenger receptor class B type I, which are overexpressed in the plaque region. Furthermore, hyaluronic acid and lovastatin-loaded nanoformulations were made with rHDL as a targeting ligand at atherosclerotic lesions. It was found that incorporating hyaluronic acid into the formulation leads to a high affinity towards the CD44 gene, which was over-expressed at the atherosclerotic plaque. Overall, the study showed the efficacy of formulations on atherosclerosis [61].

Long-chain unsaturated fatty acids or polyunsaturated fatty acids (PUFAs) are also known for their cardioprotective properties. These essential fatty acids are α-linolenic acid, long-chain ω-3 eicosapentaenoic acid, and docosahexaenoic acid [106]. Nanoemulsions loaded with synthetic short-chain ceramides, i.e., N-hexanoylsphingosine or 17-β-estradiol, significantly inhibited human arterial vascular smooth muscle cell migration and proliferation. It was observed that either alone could not elicit such activity as observed in combination. The observed anti-proliferative activity may lead to advanced restenosis therapy [62]. In one study, statin was incorporated with omega-3 fatty acids and engulfed in a nano lipid carrier (NLC) which formed micelles in the intestinal tract. This enables better absorption of statin, while omega fatty acid inhibits VLDL, CH, and TG production, lowering lipids in the biological system. Furthermore, developed NLC improved the HDL level (150 mg/dL) compared to the marketed formulation of the atorvastatin in the in vivo animal models [103]. Subsequently, PUFAs such as α-linolenic acid, docosahexaenoic acid and eicosapentaenoic acid have been incorporated into gold nanoparticles (AuNPs). This study was performed to increase the HDL concentration in the blood circulation. In vivo study in the obese rat suggested that PUFAs and PUFAs incorporated AuNPs have increased ~1.2- and 1.6-fold-higher HDL, respectively, compared to the control group [107]. A new class of nanocarrier was developed by researchers with antioxidant polymer cores, while the outer layer was decorated with scavenger receptors targeting amphiphilic macromolecules. A polyphenolic compound was chosen because of its hypolipidemic, anti-inflammatory as well as antioxidative properties. For this, ferulic acid-based poly (anhydride-ester) nanoparticles were taken to inhibit the uptake of elevated levels of oxidized LDL and to normalize ROS in macrophages. An increase in the anti-oxidizing compound will produce a negative impact. Hence, nanoparticles were used to minimize this drawback and provide cardio-protective properties. This targeted system can decrease the development of foam cells in atherosclerotic and hyperlipidemic patients [108].

#### 3.2.3. Anti-Hypertensive Nanomedicines

Hypertension is a chronic condition that causes a rise in blood pressure from normal blood pressure to an elevated blood pressure of 140/90 mm Hg. At this level, hypertension for a prolonged duration can cause irreversible damage to organs and increases morbidity and mortality. It was found that patients suffering from arterial hypertension have an escalated cardiac output or systemic vascular resistance, or both. The former is commonly seen in younger patients, i.e., cardiac output is high. While the latter is often seen in older patients, i.e., elevated systemic vascular resistance and stiffness of the vasculature [109]. The diagnosis of arterial hypertension has been of great interest. Studies have also been carried out at a genetic level called geneosensors. It was reported that theranostic DNA-AuNPs nanocomplexes were prepared and evaluated to detect hypertension at intron 16 of the ACE gene [110]. Many hypotheses are based on hypertension linked with excess ROS generation in the central nervous system. Researchers have developed chemically distinct nanoformulation complexes to curb Angiotensin-II-dependent hypertension. These were equipped with copper/zinc superoxide dismutase protein, called nanozymes [63]. Additionally, administration of vasoactive intestinal peptide (VIP), a therapeutic peptide in humans, causes potent vasodilatory effects and lowers the systemic arterial pressure. However, in free form, it is easily hydrolyzed. To prevent hydrolysis, it was encapsulated in long circulating sterically stabilized liposomes that stabilize the molecule in alpha-helix conformation, which favored conformation for peptide–receptor interactions for improved VIP bioactivity [64]. Other delivery systems for hypertensive drugs have been developed, e.g., transdermal delivery of hypertensive drugs was developed using invasomes containing phospholipids, ethanol and terpenes. An in vivo study demonstrated that developed invasomes could reduce elevated blood pressure by 20% compared to the control isradipine. This could be due to the higher permeability of the isradipine from invasomes through rat skin [65]. One conventional method of controlling hypertension was performed by administering ACE-I inhibitors orally. Most drugs in this category are effective for a few hours and require quarterly administration. These drugs need a controlled delivery system that may be achievable using nanocarriers. The developed formulation was found to produce a long-term inhibitory effect on the ACE receptor (~22 h), whereas free captopril had an inhibitory effect for up to 30 min [111]. Hence, carvedilol was administered by loading it with solid lipid nanoparticles (SLN) which increased its bioavailability by decreasing the first-pass effect. The oral bioavailability of N-carboxymethyl chitosan coated carvedilol-loaded SLN and control SLN was 3.16 ± 0.16-fold and 1.46 ± 0.11-fold higher than plain carvedilol [112]. Hence, the above studies have suggested that nanomedicine is capable of management, treatment and diagnosis of hypertension.

#### 3.2.4. Nanomedicines for Pulmonary Arterial Hypertension (PAH)

Pulmonary arterial hypertension (PAH) is a disorder in which the cross-sectional area for blood flow towards the lung is restricted [113]. PAH is identified by increased vasoconstriction and destruction of the precapillary arterioles. PAH leads to heart failure due to the chronic elevation of the pulmonary arterial pressure, which is fatal to the patient. PAH is often accompanied by other lung disorders such as asthma and chronic obstructive pulmonary disease (COPD) [114,115]. Conventional drug therapy for PAH typically includes prostacyclin agonists, endothelin receptor antagonists and nitric oxide promoters. However, the downfalls of these traditional drugs are that they have a short half-life, instability, and require a high efficacious dose. Additionally, due to their non-targeting mechanism, high chances of adverse systemic side effects have been observed in conventional PAH therapy [116]. A potent vasodilator such as sildenafil was used to change the hemodynamic in the pulmonary arteries for the betterment of the patients. The half-life was increased by encapsulating sildenafil with PLGA. It was observed that reduction in the pulmonary hemodynamics (pulmonary artery pressure and pulmonary vascular resistance index) for control sildenafil was ~90–120 min, whereas ~240 min for sildenafil PLGA nanoencapsulation [117]. A comparative study between NLCs versus SLNs for the drug sildenafil was evaluated in vitro on the A549 cell line and in vivo in rats. Due to the controlled release nature of lipid carriers, a subtle change in vascular tone over time was observed, which provided a milder effect [118]. Another nanocarrier, MIL-89 was formulated for sildenafil and showed promising vasodilatory activity. Furthermore, advanced studies are required to assess the best-suited nanocarriers for sildenafil [119]. Endogenous compounds such as prostacyclin were found to be effective vasodilators. They are released in blood vessels and binds to specific receptors such as cytosolic peroxisome proliferator-activated receptors (PPAR) and IP receptors. Once bound to these receptors, it activates and causes vasodilation with reduced thrombosis, vascular remodeling, and inflammation. However, prostacyclin drugs are limited as they need to be injected and can cause discomfort and infection at the injection site. Additionally, it causes other undesirable effects such as systemic hypotension, flushing, jaw pain, and nausea [120]. In some studies, prostacyclin and its analogs were formulated for controlled release utilizing lipid-based carriers. The controlled release was observed in the case of iloprost-loaded liposomes projecting high stability during aerosolization [121]. Secondly, plain and PEGylated transferrin-conjugated liposomes demonstrated a high level of stability after the nebulization process [122]. Similarly, it was observed that liposomes were acceptable by pulmonary tissues as they easily dissolved in the pulmonary fluid and served as surfactants when administered through the inhalation route. A controlled release nanoformulation loaded with fasudil for inhalation was developed using liposomes as a nanocarrier. The developed nanocarrier was demonstrated to have a longer vasodilatory effect up to 3 h [66]. Another potent vasodilator with anti-proliferative and anti-coagulant properties such as NO was formulated in hydrogel-like polymeric nanoparticles for releasing NO. It was found to cause systemic (aorta) and pulmonary vessel relaxation without viability on pulmonary artery smooth muscle cells [123]. In another study, an anticancer drug known as imatinib (PDGF-receptor tyrosine kinase inhibitor) was found to have antiproliferative and proapoptotic activity. Currently, this drug is not approved for PAH therapy, but researchers are trying to suppress its side effects, which are outweighed by its usefulness. For example, imatinib was encapsulated in a PLGA-based nanocarrier with fluorescein isothiocyanate (FITC) and in vivo assessment was performed on monocrotaline (MCT) induced PAH in rats. The result showed sustained antiproliferative effects and the dose was proposed as similar for the treatment of cancer to prevent undesirable side effects. Additionally, the result showed that the single intratracheal administration of phosphate-buffered saline and FITC nanoparticles elevated the right ventricular systolic pressure (RVSP) to 2.69 and 2.60 fold, respectively, as compared to control [67]. Heart failure is a significant medical and public-health issue that necessitates efficient heart failure treatment. Liu et al., recently developed a non-invasive nanotherapeutic delivery system by inhalation to alleviate heart failure. They prepared a ROS scavenging material (TPCD) that was then turned into anti-oxidative and anti-inflammatory nanoparticles (i.e., TPCD nanoparticles). A multilayered targeted TPCD nanoparticle was developed by decorating it with a mitochondrial-targeting ligand. In mice, bronchial deposition of inhaled TPCD nanoparticles was investigated. The in vivo effectiveness was assessed in mice with doxorubicin (DOX)-induced cardiomyopathy. Furthermore, the loading of peptide Ac2-26, an antioxidative, anti-inflammatory and pro-resolving nanotherapy was produced. The internalization of TPCD nanoparticles in cardiomyocytes and scavenging surplus ROS were found to reduce DOX-triggered oxidative stress and cell damage. TPCD nanoparticles were deposited in the heart of mice after passing through the vascular endothelium membranes in the lungs. On the other hand, the inhaled nanoparticles substantially prevented DOX-triggered heart failure in mice. Additionally, the targeted nanoparticles were significantly improved the heart targeting, uptake efficacy, and mitochondrial localization potential with enhanced therapeutic benefits (Figure 2C) [48]. Thus, studies have demonstrated the potential application of nanomedicine for the theranostic application in PAH.

#### 3.2.5. Nanomedicines for Stroke

**Figure 5:**
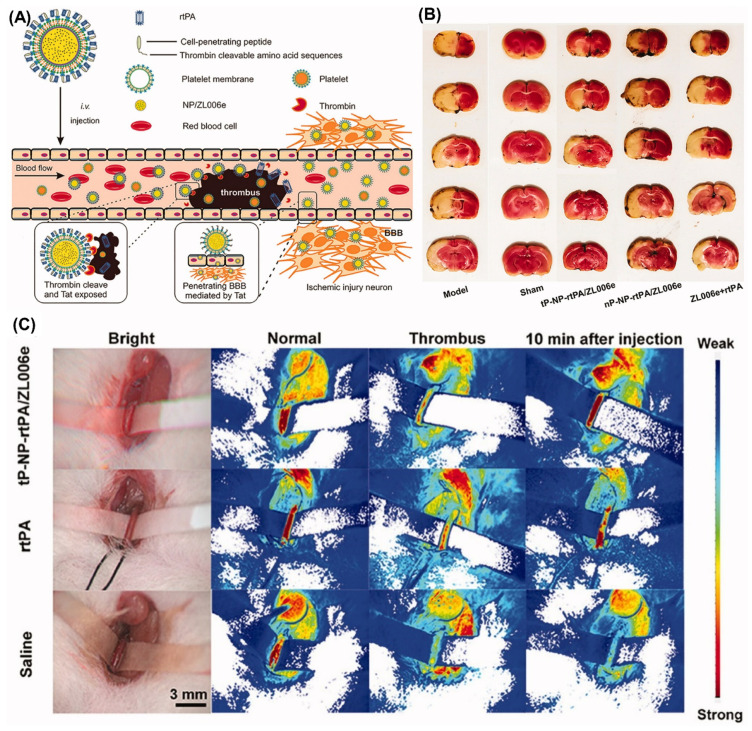
Targeted theranostic delivery of the rtPA and ZL006e loaded nanoplatelet and in vivo activity for the treatment of ischemic stroke. (**A**) Graphical representation of the targeted nanoplatelets and their release at targeted site. (**B**) Represent neuroprotective activity in the different group (nonischemic area is represented by red and infarct is represented by white). (**C**) Represented the targeted thrombolytic activity and blood stream recovery in the in vivo rat model whose carotid artery was damaged by FeCl_3_. Reproduced with permission from ref [130]. (American Chemical Society^®^ 2019).

An ischemic stroke is caused by a cardiac embolism in the blood vessels that travel to the brain. Ischemic stroke is a condition caused by abrupt loss of blood circulation to a portion of the cerebral tissue, often caused by thrombosis and embolism, leading to irreparable damage to neurological tissues and loss of motor function [124,125]. Anticoagulants are preferred therapy for the prevention of cardio-embolism recurrence. Hence, stroke therapy must begin before a cerebral infarct; hence, the early diagnosis of cardio-emboli is required [124,126]. Other treatments for stroke include neuroprotectives, small molecule thrombolytics, sonothrombolysis, perfluorocarbon nanoparticles and fibrinolytic nanoparticles [125]. The small molecule thrombolysis therapy must involve tPA administration, which transforms plasminogen into active plasmin. However, the risk of tPA outweighs its benefit [127]. Therefore, researchers have reported r-tPA loaded into polysaccharide-poly(isobutyl cyanoacrylate) nanoparticles functionalized with fucoidan on its surface for targeted therapy of thrombus. After investigation, the thrombus density was reduced to one-third of its original sizes in mice showed a more significant percentage of reduction in thrombus size [68]. Additionally, fibrin-specific thrombolytic agents, namely urokinase-loaded perfluorocarbon nanoparticles, alleviated acute cerebral ischemia by reducing 40% of clot volume after 30 min of administration. It was also proposed to be an alternative to r-tPA with significant vascular constraint, a simple administration protocol and lessenzyme exposure [69]. In one study, nitroxide radicals encapsulated in nanocarriers were effectively accumulated in the infarct region, leading to higher availability and higher free radical scavenging properties [128]. In another study, cerium oxide nanoparticles (CNP) demonstrated the potential to reduce cerebral ischemia and provided neuroprotection. In the rodent stroke model, the PEG coated CNP was used in the concentration of 0.5–0.7 mg/kg bodyweight, which showed a decrease in 50% of brain infarct compared to the control [129]. Recently, Xu et al., developed a bioengineered theranostic nanoplatelet for targeted delivery of r-tPA and neuroprotectant (ZL006e) for the treatment of ischemic stroke. Tat-peptide was used as a targeting agent that was cleaved by upregulated thrombin and promoted the targeted release. The in vivo animal study in the rat demonstrated 63 and 72% lower ischemia area and ROS by targeted nanoplatelets than free drug. In vivo neuroprotective activity, targeted thrombolytic and blood stream recovery of the developed nanoplatelets are presented in Figure 5 [130]. The developed nanomedicine modalities were able to address the challenges such as (i) gap between the prevention and therapy (ii) risk factors (iii) personalized therapy and (iv) need of first line treatment and personalized care of CVRDs.

## 4. An Update: Nanomedicine in Clinical Trials for CVRDs

Clinical trials for nanoparticles based on the intervention of CVRDs were searched at clinicaltrials.gov using keywords such as “liposomes,” “nanoemulsions”, “quantum dots”, “dendrimers”, “polymeric nanoparticles” etc. The better-advanced search combination keywords such as “cardiovascular diseases”, “vascular disease” or individual CVRDs disorders were also used. The results suggested that nanoparticles were helpful for visualization of inflammation and diagnosis of acute CVRDs using MRI. Additionally, prevention of restenosis and plasmonic photo-thermal therapy of atherosclerotic plaques were observed [131]. Many trials are still in their initial phases. However, few have completed their clinical trials using iron oxide nanoparticles. Sinerem^TM^ is the contrasting agent in MRI that uses ultrasmall superparamagnetic iron oxide (USPIO) particles that were clinically shown to increase accumulation in macrophages surrounding the inflamed plaques [132]. Moreover, asymptomatic patients were also kept under clinical observation [133]. A comparative study was performed and the differences between symptomatic and asymptomatic patients were observed for the number of quadrants and signal peaks [134]. Similarly, USPIO used in MRI scans was helpful in the detection of inflammation-induced during acute myocardial infarction in the primary clinical experiment [135]. Some approval of clinical trials were made to mitigate inflammatory atherosclerosis employing good manufacturing practice (GMP) grade liposomes containing prednisolone phosphate [136]. The first clinically studied liposomal prednisolone in inflammatory atherosclerosis was found to have a better pharmacokinetic profile in humans without any anti-inflammatory effect. Instead, it paved the way for imaging-assisted technology [93]. A clinical trial involving multiple interventions and plasmonic photothermal theranostic treatment was observed in atherosclerotic subjects. The subjects were divided into groups and each group was given one intervention of silica gold nanoparticles which were bioengineered on artery patch or silica-gold iron-bearing nanoparticles and attached with micro-bubbles for target delivery. Lastly, stem cells were compared using magnetic navigation and stent implantation systems. The trial was carried out for a year and resulted in significant regression of atheroma size in silica gold plasmonic photothermal therapy. The clinical outcome showed a significantly minor risk of cardiovascular death in nano group 91.7% compared to other group, i.e., ferro group and stenting control group 81.7% and 80%, respectively [137]. Novel compound, i.e., LABR-312, is undergoing clinical trial on subjects with coronary narrowing to decrease the risk of restenosis. This trial involved the methodology of the biorest liposomal alendronate with stenting study (BLAST) that evaluates the safety and efficacy of this compound. Results were highly acknowledged in proinflammatory state subjects suffering from diabetes mellitus [138].

## 5. Latest Developments in Targeted Nanomedicines for CVRDs

A milestone in advanced therapy for CVRDs will be the involvement of artificial intelligence to keep up with the fast pace advancement of cancer therapy, which involves the application of nanomedicine in conjunction with artificial intelligence [139]. In recent gene therapy, a virion genetic carrier was utilized to treat myocardial infarction. It was used to transport genetic material to the infarct site and transport stem cells to cardiomyocytes for regeneration and repair [140]. Other cardiomyocyte generation therapies include stem cell therapy, which involves the application of mesenchymal stem cells that showed promising results in the treatment of CVRDs. However, the engraftment of stem cells into cardiomyocytes has proved to be a challenging feat due to inefficient cell binding. The cell binding capacity was enhanced with externally triggered targeted drug delivery incorporating a magnetic field. Furthermore, numerous studies utilizing magnetically targeted nanoparticles include ferumoxytol and superparamagnetic oxide nanoparticles incorporated with human-derived and rat-derived mesenchymal stem cells, respectively [141,142]. In one study, cellular regeneration of cardiomyocytes was enhanced by insulin-like growth factor-1 incorporated into biotinylated peptide nanofibers [143].

Other nanomedicines that exhibit potential for future therapies are aptamers, DNA oligonucleotides and exosomes that transport protein, mRNA, and miRNA [144]. Another direct method of cardiac fibroblast reprogramming has been reported, including the encapsulation of microRNAs in PLGA-polyethyleneimine nanocarriers. Hence, these nanospheres provided alternative non-viral gene delivery and therapy [145,146]. Tissue engineering is another advanced technique employed in tissue regeneration and cellular repair. Biodegradable nanopolymers were employed in cardiac tissue engineering in implantable artificial blood vessels, injectable gels, and cardiac patches. Synthetic nanopolymer-made scaffolds can act like outer layer cells texture, mechanical, and electrical properties of cardiomyocytes, which can be used alternatively in the infarcted area, to stimulate cardiovascular tissue growth [147]. Other studies suggested a different pathology of CVRDs, i.e., the relationship between gut microflora andCVRDs development, indicating a better understanding and potentially newer targets for CVRDs. A commonly used and extensively researched compound such as curcumin was formulated to achieve active targeting in CVRDs and showed improved cardioprotective properties due to its anti-inflammatory attributes [148]. In the past few decades, coronary stents have been clinically used, and their modified and advanced versions, such as drug-eluting stents (DES), have been reported. The DES was incorporated with a nanoparticle drug that provided a targeting therapy at the insertion site. In coronary stent, various anti-proliferators such as imatinib mesylate, pitavastatin, paclitaxel, clodronate and lisinopril were associated, which prevented the proliferation of VSMC. These anti-proliferators were delivered via nanocarriers from the coronary stents, which prevented restenosis [149,150,151].

Although nanoparticles have a lot of promise for treating atherosclerosis, there are still some difficulties in translating them into clinical practice. The lack of animal research to translate them into clinical trials for human treatment is a serious challenge, which could be attributed to the fact that animal models cannot accurately mimic atherosclerotic progression in humans. The long-term stability and effectiveness of nanomedicine must be thoroughly assessed.

## 6. Challenges in the Translation of Nanomedicine for CVRDs

The concept of active targeting in nanomedicine has proven to be a challenging feat in treating CVRDs. It has a complex etiology and pathophysiology, and many intricate mechanisms are behind CVRDs [152]. Moreover, the small dimension of nanocarriers created a nuisance when being opsonized by macrophages. Nanomedicines are identified as foreign objects that are quickly engulfed and inactivated by the mononuclear phagocyte system, preventing them from concentrating in targeted cells and tissues. PEGylation of nanocarriers solved this problem by utilizing PEG on the surface of nanocarriers. PEGylation inhibits opsonization and phagocytosis, increasing its plasma life span [153]. However, regardless of its benefits, PEGylation may have some limitations in the form of a triggered immune system that causes antibody production that binds to PEG and increases its clearance at a fast pace [154].

Since pre-clinical and clinical trials occupy a lengthy period, nanomedicine launching into the marketing sector is at a snail pace [155]. In light of the regulatory bodies, nanomedicine has been categorized in similar categories to other formulations in regard to pre-clinical and clinical trials when it requires FDA approval, i.e., Investigational New Drug (IND) and New Drug Application (NDA) [156]. Ongoing regulatory and newer generation gaps have made uneven progress in marketing newer nanomedicine [157]. The safety issue is another concern in the clinical application of nanomedicine. Specific shapes of nanocarriers, such as cylindrical tubes of carbon nanotubes have raised safety concerns and toxicity issues. For instance, carbon nanotubes were found to be producing mesothelioma, which was similar to asbestos induced carcinogenesis. Similarly, 1.4 nm gold nanoparticles are toxic, whereas 15 nm gold nanoparticles are nontoxic. Additionally, in some studies silver and iron oxide nanoparticles have been found to be toxic both in vitro and in vivo. The harmful effects of these nanoparticles are mainly due to the generation of reactive oxygen species, disruption of cellular components and immunological reactions [158]. Nonetheless, the evaluation of toxicity profiles of nanomedicine has been a challenge, specifically with in vivo assessment and long-duration toxicity studies [159,160]. Nanomedicine approved by the FDA or under clinical trials for diagnosis or therapy of CVRDs is presented in Table 3.

## 7. Future Perspective

In the near future, a breakthrough in targeted nanomedicine concerning CVRDs will be witnessed in the global health sector. Non-invasive diagnostic and therapeutic treatments will lead to better patient compliance and a non-surgical approach. Furthermore, better advancement in cellular regeneration in the myocardial infarcted area may be available to study cellular growth. This process requires sophisticated targeted nanocarriers incorporating stem cells into the affected area. Modification of genes may also be seen as an alternative treatment. Nanomedicines will carry genetic material in the targeted genetic sequence in myocardial cells or vessels. With external stimuli being applied, such as a change in pH or utilizing a magnetic field, controlled and sustained targeted nanomedicine can be achieved with good feedback from in vitro and in vivo studies.

Other advancements in artificial intelligence will have a predictive outcome and will be in silico methods of nanomedicine evaluation. Additionally, better improvement in the solubility profile of drugs using nanomedicines will lead to better efficacy of low soluble drugs. Safety and efficacy are essential in the acceptance and clinical application of theranostic nanomedicine in CVRDs. Such progression will lead to a lower mortality rate in CVRDs. Moreover, theranostic nanomedicine for CVRDs will be approved and will soon reach the global market for efficacious therapy.

## 8. Conclusions

Nanomedicines can transport drugs, active moieties, and genetic materials through passive and active targeted delivery mechanisms. Advancements in nanomedicines have paved a better way for the therapy of acute and chronic CVRDs. Further development in molecular imaging will boost the diagnostic and theranostic efficacy of nanomedicines. Mainly, clinical aspects must be focused on for speedier approval of theranostic nanomedicine. As many CVRDs are interrelated, the causative source must be thoroughly understood, and discoveries of newer targets could be lifesaving. Polypharmacy in CVRDs can cause undesired side effects; a targeted and prolonged effective medication is required in the future for such multifaceted diseases. Atherosclerosis and myocarditis are the most prevalent CVRDs requiring a proper and suitable diagnostic mechanism for their treatment and mitigation. Hence, theranostic nanomedicines can provide a controlled and targeted form of diagnosis and therapy for CVRDs with minimal side effects.

## Figures and Tables

**Table 1:** Different targeted/theranostic approaches of nanomedicine for CVDs.

Diseases/CVRDs	Drug/Nanocarrier	Targeted Areas	Treatment Time	Tested Doses and Route of Administration	Inferences	Ref.
Angina pectoris	Ivabradine/Polymeric nanoparticles	Funny channels of SA-node	3 days	1.54 mg/kg, 2 mL; oral	Increased permeability; anti-anginal effects lasted for 3 consecutive days.	[40]
Angina pectoris	Verapamil/NLCs	α-adrenergic receptors of myocardial cells	24 h	-	Prolonged drug release; higher cellular uptake.	[41]
Myocarditis	Iron metal/nanoparticle, Materials InstituteLavoisier-89	Artery endothelial and smooth muscle cells	24 h	-	Shown anti-inflammatory effect and reduced chemokine CXCL8.	[42]
Myocardial infarction	Magnetic nanoparticles	Extracellular matrix—Metalloproteinase inducer	-	50 mg/Kg i.v.	In vivo visualization and regression of acute myocardial infarction.	[26]
Myocardial infarction	miR199a-3p/macrophage membrane coated nanoparticles	IL-1β, -6, and tumor necrosis factor alpha (TNF-α)	2 weeks	2.0 mg/kg; i.v.	Reduction in inflammatory cells and increased cell proliferation abilities.	[43]
Myocardial infarction	Pioglitazone/Poly (lactic acid/glycolic acid) nanoparticles	Peroxisome proliferator-activated receptor-gamma (PPARγ)	3 days	1.0 mg/kg; i.v.	Suppression of Ly6C^high^ inflammatory monocyte and inflammatory gene expression.	[44]
Myocardial infarction	Oleate adenosine prodrug-atrial natriuretic peptide/Lipid nanocarriers	Natriuretic peptide receptors of ischemic heart	48 h	1 mL per rat; i.v.	Reduction in infract size.	[45]
Myocardial infarction	Salvianolic acid B-ginsenoside Rg 1/Lipid-polymer hybrid nanoparticles	α_v_β_3_ integrin receptor	3 days	Sal B: 10 mg/kg, PNS: 10 mg/kg; i.v.	Reduction in infract size.	[46]
Myocardial infarction	Radix Ophiopogonis polysaccharide/Mono polyethylene glycol	Hypoxic tissues of the heart	4 days	4 μmol/kg; i.v.	Increased drug accumulation in the infarcted myocardium.	[47]
Heart failure	ATTPCD bioactive nanoparticles	Pulmonary circulation-mediated heart targeting	12 to 48 h	50 mg/kg	Prevention of heart failure and imaging of heart and vital organ.	[48]
Thrombosis	Streptokinase/Liposome-encapsulated & microencapsulated	Plasminogen	2 h	6000 IU/kg; i.v.	Lesser cases of occlusions were observed.	[49]
Thrombosis	Streptokinase/platelet-derived Microparticles—inspired nanovesicles	Plasminogen	Overnight	30 mg/kg; i.v.	Relieving thrombolytic payload.	[50]
Thrombosis	_D_-phenylalanyl-_L_-prolyl-_L_-arginyl chloromethyl ketone/Semipermeant perfluorocarbon core nanoparticles	Thrombin	1 month	-	Inactivates thrombin.	[51]
Thrombosis	tPA/Chitosan magnetic nanoparticles	Fibrin clot	2 h	150 μL; i.v.	Increased efficacy of drug.	[52]
Thrombosis	Recombinant tissue plasminogen activator/magnetofluorescent nanoparticle	Thrombus clot	1 h	14 mg/kg; i.v.	Exhibits theranostic capabilities and high affinity towards clot.	[53]
Thrombosis	Fibrin targeted H_2_O_2_-responsive nanoparticles	Fibrin	3 min	24 μg/kg i.v.	Image obstructed vessels and inhibit thrombus formation.	[54]

NLCs: Nanostructured lipid carriers; IL: Interleukin; tPA: Tissue plasminogen activator.

**Table 2:** Different targeted/theranostic approaches of nanomedicine for CVRDs.

Diseases/CVRDs	Drug/Nanocarrier	Targeted Areas	Treatment Time	Tested Doses and Route of Administration	Inferences	Ref.
Atherosclerosis	Andrographolide/PEG-poly(propylene sulphide) micelles	NF-κB signaling pathway	30 days	45 μg/g micelle, 2 μg/g; andro i.v.	Increased delivery efficiency.	[55]
Atherosclerosis	Prednisolone/Liposomes	Atherosclerotic macrophages	10 days	1.5 mg/kg; i.v.	No anti-inflammatory effect seen.	[56]
Atherosclerosis	IL-10/Arginylglycyl aspartic acid conjugated pluronic-based nanocarriers	Atherosclerotic plaques	1–3 weeks	1.05 mg of NC with 5 μg IL10; i.v.	Could inhibit the progression of atherosclerotic plaques.	[57]
Atherosclerosis	Fumagillin/Paramagnetic nanoparticles	Endothelial αvβ3 integrin	2–4 h	1.0 mL/kg; i.v.	Quantification and inhibition of angiogenesis.	[58]
Atherosclerosis	PLGA nanoparticles	plaque-targeted peptides PP1 and cRGD	6 h	-	Diagnosis and therapy of advanced atherosclerotic plaques.	[59]
Hyperlipidemia	Simvastatin/chitosan NPs	HMG-CoA reductase enzyme	16 weeks	10 mg/kg; Oral	Increased hypolipidemic effect.	[60]
Hyperlipidemia	Lovastatin/Hyaluronic acid-reconstituted high-density lipoprotein	Atherosclerotic lesions	8 weeks	2 mg/kg; i.v	Greater atheroprotective efficacy.	[61]
Hyperlipidemia	N-hexanoylsphingosine or 17-β-estradiol/Nanoemulsions	MAPK enzyme	24 h	-	Greater anti-proliferative activity.	[62]
Hyperlipidemia	Copper/Zinc superoxide dismutase/Poly-L-lysine (PLL_50_)-polyethylene glycol block co-polymer	Central nerves	9 days	130–150 U CuZnSOD activity; ICV injection	Stabilized angiotensin-II-dependent hypertension.	[63]
Hyperlipidemia	Human vasoactive intestinal peptide (VIP-α)/Liposomes	Cognate receptors of vascular smooth cells	6 h	0.5 mL; i.v	Potent vasodilation and lowers systemic arterial pressure.	[64]
Hyperlipidemia	Isradipine/Invasomes	L-type calcium channels of vascular smooth muscle and myocardium	24 h	Transdermal flux	Improved the antihypertensive activity.	[65]
Pulmonary arterial hypertension	Fasudil/Liposomes	Rho-kinase receptors	4 weeks	3 mg/kg; intratracheal	Prolonged vasodilatory effect for three hours.	[66]
Pulmonary arterial hypertension	Imatinib/Polylactide-glycolide nanoparticles-fluorescein isothiocyanate	Platelet–derived growth factor receptors	3 weeks	1 mg/kg; intratracheal instillation	Sustained antiproliferative effects.	[67]
Stroke	rtPA/Polysaccharide-poly(isobutylcyanoacrylate)-fucoidan nanoparticles	P-selectin	30 min	2.5 mg/kg; i.v.	Thrombus density reduced to one-third of its original sizes.	[68]
Stroke	Urokinase anti-fibrin monoclonal antibodies/ Perfluorocarbon nanoparticles	Fibrin clot	2 h	2 mL/kg; i.v.	Alternative to reconstituted-Tissue plasminogen activator.	[69]

PEG: Poly(ethylene glycol); IL-10: Interleukin-10; MAPK: Mitogen-activated-protein-kinase; rtPA: Recombinant tissue plasminogen activator.

**Table 3:** List of nanomedicine approved by FDA or presently in clinical trial for diagnosis or therapy of CVRDs.

Drug/Nanocarrier	Clinical Trial Types	Purpose	Indications	Benefits	Ref.
USPIONPs	N/A	Diagnostic	Atherosclerotic plaque	Noninvasive method of imaging carotid atheroma.	[132,133,134]
USPIONPs	N/A	Diagnostic	Acute myocardial infarction	Noninvasive method of imaging inflammatory myocytes.	[135]
Prednisolone phosphate/liposomes	Randomized, placebo-controlled study	Therapeutic	Inflammatory atherosclerosis	Benefiting image assisted technology	[136]
Silica-AuNPs	Multi-center, observational, open-label, three arms study	Therapeutic	Atherosclerosis	Application of novel invasive Plasmonic photothermal therapy using near-infrared laser irradiation.	[137]
LABR-312/Liposomes	Prospective, randomized, multicenter, double-blind, placebo-controlled trial	Therapeutic	Atherosclerosis	Much more effective in subjects with proinflammatory state, such as diabetes mellitus and high baseline monocyte count.	[138]
TriCor/Nanocrystals	FDA approved	Therapeutic	Hyperlipidemia	Tablets for oral use is available.	[161]

USPIONPs: Ultrasmall super paramagnetic iron oxide nanoparticles; AuNPs: Gold Nanoparticles.

## Data Availability

Not applicable.
